# Evaluation of HLA-DQ2 and HLA-DQ8 haplotypes in patients with endometriosis, A case-control study

**DOI:** 10.1016/j.clinsp.2023.100317

**Published:** 2024-03-02

**Authors:** Marina P. Andres, Alessandra Peloggia, Henrique M. Abrao, Thais F. Magalhaes, João Siufi Neto, Mauricio Simões Abrão

**Affiliations:** a Divisão de Clínica Ginecológica, Departamento de Obstetrícia e Ginecologia, Hospital das Clínicas, Faculdade de Medicina, Universidade de São Paulo (HCFMUSP), São Paulo, SP, Brazil; b Divisão de Clínica Ginecológica, BP ‒ A Beneficência Portuguesa de São Paulo, São Paulo, SP, Brazil; c Centro de Pesquisa em Saúde Reprodutiva de Campinas (CEMICAMP), Campinas, SP, Brazil

**Keywords:** Endometriosis, Celiac Disease, HLA-DQ2, HLA-DQ8, Anti-tissue transglutaminase IgA

## Abstract

**Objective:**

To evaluate the relationship between genetic haplotypes associated with celiac disease (Human Leucocyte Antigen [HLA] DQ2 and DQ8) with the diagnosis, clinical presentation, and location of endometriosis in Brazilian women.

**Method:**

A retrospective cross-sectional study, was conducted in a Tertiary hospital.

**Patients:**

Women aged 18–50 years who underwent HLA-DQ2 and HLA-DQ8 haplotype analysis.

**Intervention:**

The patients were divided into endometriosis and control groups and evaluated for symptoms; endometriosis location, American Society for Reproductive Medicine (ASRM) stage, and the presence of anti-tissue transglutaminase IgA (anti-TgA), HLA-DQ2, and HLA-DQ8 markers.

**Results:**

A total of 434 consecutive patients with (*n* = 315) and without (*n* = 119) endometriosis were included. Pain and infertility were more frequent in the endometriosis group than in the control group. The presence of HLA-DQ2, HLA-DQ8, and anti-TgA was similar between both groups. The presence of HLA-DQ2 and HLA-DQ8 markers did not differ based on age, pain symptoms, ASRM stage, or endometriosis location.

**Conclusion:**

Although there are similarities in inflammatory markers and pathophysiology between celiac disease and endometriosis, this study found no significant associations in the presence of HLA-DQ2 or HLA-DQ8 haplotypes and endometriosis.

## Highlights

• Celiac disease and endometriosis share inflammatory markers and pathophysiology.
• HLA-DQ2 and/or HLA-DQ8 were similar between the control and endometriosis groups.
• Anti- transglutaminase igA was similar between the control and endometriosis groups.
• HLA-DQ2 and DQ8 had no difference in age, symptoms, endometriosis stage or location.

## Introduction

Celiac Disease (CD) is an autoimmune disorder caused by T-cell reactivity to gluten consumption. It is characterized by small intestine inflammation, villous atrophy, and crypt hyperplasia. CD can also cause systemic symptoms in approximately half of the affected patients.^1^^,^^2^ The pathophysiology of CD is complex and involves both environmental and intrinsic variables. Predisposing genetic factors, specifically certain Human Leukocyte Antigen (HLA) haplotypes, are necessary but not sufficient for the symptoms to appear.^2^ The HLA-DQ2 haplotype, coded by the genes HLA-DQA1*0501 and HLA-DQB1*0201, is strongly associated with CD and found in up to 95 % of patients. The remaining patients carry either HLA-DQ8 or a variant of HLA-DQ2.^2^, ^3^, ^4^

HLA-DQ molecules present gluten peptides to T-cells, leading to inflammation and cytokine production.^5^ Genome-wide association studies have observed that the HLA region, particularly the DQA1 and DQB1 genes, has the strongest risk of CD.^6^^,^^7^ These genetic loci have also been observed in several immune-mediated diseases, suggesting that CD shares common pathways with other chronic diseases such as endometriosis.^6^, ^7^, ^8^

The diagnosis of CD relies on a combination of clinical, laboratory, and anatomopathological findings. It is confirmed by alterations seen in duodenal biopsy samples alongside positive antibody testing for anti-endomysial, anti-tissue transglutaminase (anti-Tg), or anti-gliadin antibodies.^9^ The risk of CD in HLA-DQ2 and HLA-DQ8 carriers ranges from 0.17 % to 12.8 %.^10^,^11^ At the population level, disease prevalence can be estimated by the presence of high titles of anti-Tg, while the absence of specific HLA-DQ variants suggests a negative predictor of CD.^12^

Endometriosis is defined by the presence of endometrial tissue outside the uterine cavity and affects 10 %‒15 % of women of reproductive age.^13^,^14^ The pathophysiology of endometriosis involves immunological, inflammatory, and genetic factors, some of which overlap with pathways seen in CD.^2^^,^^14^, ^15^, ^16^ Studies have shown a higher prevalence of CD in women with endometriosis compared to healthy controls, and gluten-free diets have been shown to alleviate endometriosis symptoms.^17^, ^18^, ^19^

To investigate the possible relationship between CD and endometriosis, this study aimed to assess the relation between HLA-DQ2 and HLA-DQ8 haplotypes and the diagnosis, clinical presentation, and location of endometriosis in Brazilian women.

## Material and methods

This case-control study was conducted following the STROBE Statement and included 434 consecutive women aged 18‒50 years from the Gynecologic Department of Beneficiência Portuguesa Hospital and Clinica Medicina da Mulher from 2010 to 2020 who underwent the evaluation of HLA-DQ2 and HLA-DQ8 haplotypes. The patients were divided into two groups: women with endometriosis and women without evidence of endometriosis, as determined by transvaginal ultrasound since the group has a high level of expertise in diagnosing the disease through this exam. As this was a retrospective study and the first to investigate the relation between endometriosis and HLA-DQ2 and HLA-DQ8, no sample size calculation was performed. The authors included all patients with HLA genotyping evaluated during the study period.

Data obtained from the medical records included age, pain symptoms (evaluated using the 0‒10 visual analogic scale), presence of anti-tissue transglutaminase IgA (anti-TgA), HLA-DQ2, and HLA-DQ8 markers and presence or absence of endometriosis according to transvaginal ultrasounds performed using the protocol defined by Goncalves et al.,^20^,^21^ location of lesions, and stage of endometriosis according to the American Society of Reproductive Medicine (ASRM) classification.^22^ The exclusion criteria for patients were unavailability of data and the presence of other inflammatory bowel diseases, pregnancy, or pelvic malignancies.

### Ethics

This study was approved by the Internal Review Board of the Hospital Beneficência Portuguesa of São Paulo (number 3340139/2019). Data for this study was obtained retrospectively and anonymously from medical charts. Written consent was not obtained for this study.

### Statistical analysis

The results are expressed as mean and standard deviation for normally distributed variables and as numbers and percentages for categorical variables. The Kolgomorov-Smirno was performed to evaluate the distribution of the variables. The categorical data was compared using the Pearson Chi-Square or Fisher's exact tests. Unpaired Student's *t*-test was used to compare continuous parametric variables and the Mann-Whitney *U* test was used to compare non-parametric variables between different groups. Multiple logistic regression and odds ratio analysis were used to assess the combined relationship between symptoms and the presence of endometriosis with age, HLA-DQ2, HLA_DQ8, and Anti-tissue transglutaminase IgA. Statistical significance was set at *p* < 0.05.

## Results

A total of 315 patients with endometriosis and 119 without endometriosis were included in this study. The patient characteristics and symptoms are summarized in Table 1. The mean age was 39.1 years in the control group and 36.9 years in the endometriosis group (*p* = 0.05). Patients in the endometriosis group presented with more dysmenorrhea (*p* < 0.001), deep dyspareunia (*p* < 0.001), non-cyclic pelvic pain (*p* < 0.001), cyclic dysuria (*p* < 0.001), and more infertility (58.0 % vs. 31.6 %; *p* < 0.001) than those in the control group.

**Table 1:** Characteristics of women with and without endometriosis. Parametrical variables were compared using T-Student test^a^. Categorical variables were compared using Pearson's Chi-Squared test^b^ or Fisher's Exact test^c^.

	**Control** **(***n* = **119)**	**Endometriosis** **(***n* = **315)**	
**Characteristics**	**Mean ± SD**	**Mean ± SD**	**p**
**Age (years)**	39.1 ± 10.8	36.9 ± 7.3	0.05^a^
**Dysmenorrhea (VAS)**	4.5 ± 3.9	7.1 ± 3.0	<0.001^a^
**Chronic pelvic pain (VAS)**	1.9 ± 3.1	3.6 ± 3.8	<0.001^a^
**Deep dyspareunia (VAS)**	2.3 ± 3.4	4.2 ± 3.6	<0.001^a^
**Cyclic dysuria (VAS)**	0.4 ± 1.7	0.6 ± 2.0	0.132^a^
**Cyclic dyschezia (VAS)**	1.0 ± 2.6	2.7 ± 3.7	<0.001^a^
	**Control** **(***n* = **119)**	**Endometriosis** **(***n* = **315)**	**p**
	**n (%)**	**n (%)**	
**Infertility**	24 (31.6)	112 (58.0)	<0.001^b^
**ASRM stage**			
Not Reported	‒	84 (26.7)	
I	‒	75 (23.8)	
II	‒	61 (19.3)	
III	‒	33 (10.4)	
IV	‒	62 (19.6)	
**Endometriosis type**	‒		
Not reported	‒	6 (1.9)	
Superficial	‒	77 (24.4)	
Ovarian	‒	17 (5.4)	
Deep	‒	215 (68.2)	
**Site of deep endometriosis:**			
Retrocervical	‒	201 (63.8)	
Bowel	‒	137 (43.5)	
Bladder	‒	12 (3.8)	
**Anti-tissue transglutaminase IgA, n (%)**	5 (5.2)	7 (2.9)	0.329^c^
**HLA-DQ2, n (%)**	48 (40.3)	111 (35.4)	0.337^b^
**HLA-DQ8, n (%)**	15 (12.6)	47 (15.0)	0.531^b^
**HLA-DQ2 or DQ8, n (%)**	60 (50.4)	148 (46.9)	0.522^b^

Data shown as mean ± Standard Deviation (SD); VAS, Visual Analogue Scale; ASRM, American Society for reproductive Medicine, 1996.

In the endometriosis group, the patients were classified according to the ASRM classification:^22^ Stage I in 32.5 % (*n* = 75), Stage II in 26.4 % (*n* = 61), Stage III in 14.3 % (*n* = 33), and Stage IV in 26.8 % (*n* = 62) of cases. The type of endometriosis was superficial in 24.4 % (*n* = 77), ovarian in 5.4 % (*n* = 17), and deep endometriosis in 68.2 % (*n* = 215) of patients. The main sites affected by deep endometriosis were the retrocervical (63.8 %, *n* = 201), bowel (43.5 %, *n* = 137), and bladder (3.8 %, *n* = 12).

Comparison of CD markers in the endometriosis and control groups showed no significant differences. Anti-tissue transglutaminase IgA was positive in 5.2 % of patients in the control group and in 2.9 % of patients in the endometriosis group (*p* = 0.329). There was no difference in the prevalence of HLA-DQ2 (40.3 % vs. 35.4 %; *p* = 0.0337) or HLA-DQ8 (12.6 % vs. 15.0 %; *p* = 0.531) between the control and endometriosis groups.

While analyzing the patients with endometriosis, the mean age was similar for those with and without HLA-DQ2 (37.4 ± 5.9 years vs. 36.8±7.5 years; *p* = 0.337, respectively) and HLA-DQ8 (37.4 ± 5.9 vs. 36.8 ± years; *p* = 0.477). Endometriosis symptoms such as dysmenorrhea, acyclic pain, deep dyspareunia, cyclical dysuria, cyclical dysphasia, and infertility were not significantly different between patients with or without HLA-DQ8 or HLA-DQ2. The location of endometriosis implants was also not associated with the presence or absence of HLA-DQ2 or HLA-DQ8 (Tables 2 and 3).

**Table 2:** Characteristics of women with endometriosis by HLA-DQ2 status. Parametrical variables were compared using T-Student test^a^. Categorical variables were compared using Pearson's Chi-Squared test^b^ or Fisher's Exact test^c^.

	**HLA-DQ2+** **(***n* = **111)**	**HLA-DQ2-** **(***n* = **203)**	
**Characteristic**	**Mean ± SD**	**Mean ± SD**	***p***
**Age (years)**	36.0 ± 7.4	37.3 ± 7.2	0.181^b^
**Dysmenorrhea (VAS)**	7.4 ± 2.7	6.9 ± 3.2	0.230^b^
**Acyclic pain (VAS)**	3.9 ± 3.8	3.5 ± 3.8	0.469^b^
**Deep dyspareunia (VAS)**	4.1 ± 3.8	4.2 ± 3.5	0.880^b^
**Cyclic dysuria (VAS)**	0.8 ± 2.1	0.6 ± 1.9	0.766^b^
**Cyclic dyschezia (VAS)**	2.9 ± 3.9	2.6 ± 3.6	0.488^b^
	**n (%)**	**n (%)**	
**Infertility**	38 (62.3)	74 (56.1)	0.415^a^
**ASRM stage**			
I	29 (33.0)	46 (32.4)	0.969^a^
II	22 (25.0)	39 (27.5)	
III	12 (13.6)	20 (14.1)	
IV	25 (28.4)	37 (26.1)	
**Endometriosis type**			
Superficial	30 (39.0)	47 (61.0)	0.788^a^
Ovarian	6 (35.3)	11 (64.7)	
Deep	74 (34.4)	140 (65.1)	
**Site of deep endometriosis**			
Retrocervical	68 (61.8)	132 (67.3)	0.329^a^
Bowel	46 (42.2)	90 (46.4)	0.482^a^
Bladder	5 (4.6)	7 (3.7)	0.762^c^

Data shown as mean ± SD;

a Chi-Squared test;

b T-Student test; ^c^Fisher's Exact test; VAS, Visual Analogue Scale; ASRM, American Society for Reproductive Medicine, 1996.

**Table 3:** Characteristics of women with endometriosis by HLA-DQ8 status. Parametrical variables were compared using T-Student test^a^. Categorical variables were compared using Pearson's Chi-Squared test^b^ or Fisher's Exact test^c^.

**Characteristic**	**HLA-DQ8+** **(***n* = **47)**	**HLA-DQ8-** **(***n* = **267)**	**p**
	**Mean ± SD**	**Mean ± SD**	
**Age (years)**	37.4 ± 5.9	36.8 ± 7.5	0.477^b^
**Dysmenorrhea (VAS)**	6.9 ± 3.0	7.1 ± 3.1	0.453^b^
**Acyclic pain (VAS)**	3.9 ± 4.1	3.6 ± 3.8	0.673^b^
**Deep dyspareunia (VAS)**	3.8 ± 3.5	4.2 ± 3.6	0.434^b^
**Cyclic dysuria (VAS)**	0.6 ± 1.9	0.7 ± 2.0	0.595^b^
**Cyclic dyschezia (VAS)**	3.5 ± 3.9	2.5 ± 3.7	0.120^b^
	**n (%)**	**n (%)**	
**Infertility**	16 (55.2)	96 (58.5)	0.735^a^
**ASRM stage**			
I	11 (29.7)	64 (33.2)	0.970^a^
II	10 (27.0)	51 (26.4)	
III	5 (13.5)	27 (14.0)	
IV	11 (29.7)	51 (26.4)	
**Endometriosis type**			
Superficial	12 (15.6)	65 (84.4)	0.599^a^
Ovarian	1 (5.9)	16 (94.1)	
Deep	33 (15.3)	181 (84.7)	
**Site of deep endometriosis**			
Retrocervical	32 (69.6)	168 (64.6)	0.515^a^
Bowel	23 (51.1)	113 (43.8)	0.363^a^
Bladder	2 (4.4)	10 (3.9)	0.699^c^

Data shown as mean ± SD;

a Chi-squared test;

b T-Student test;

c Fisher's exact test; VAS, Visual Analogue Scale; ASRM, American Society for Reproductive Medicine, 1996.

Multiple logistic regression was performed (Table 4) and showed no significant relation between pain symptoms, infertility, presence of endometriosis with the presence of HLA-DQ2 and HLA-DQ8 haplotypes, anti-tissue transglutaminase IgA, and age.

**Table 4:** Multiple regression analysis evaluating symptoms, the presence of endometriosis, and HLA-DQ2, HLA-DQ8, age, and Anti-tissue transglutaminase IgA.

Variable	Co-variable	OR	95% IC		p^a^
			Inferior	Superior	
Dysmenorrhea	Anti TGA	0.548	0.132	2.267	0.406
	HLA-DQ2	1.168	0.717	1.904	0.532
	HLA-DQ8	1.286	0.659	2.509	0.460
	Age	0.931	0.902	0.961	0.000
Dyspareunia	Anti TGA	0.902	0.255	3.186	0.872
	HLA-DQ2	0.740	0.456	1.203	0.224
	HLA-DQ8	0.793	0.407	1.546	0.496
	Age	0.992	0.963	1.023	0.621
Dyschezia	Anti TGA	347664436.454	0.000		0.999
	HLA-DQ2	0.995	0.542	1.830	0.988
	HLA-DQ8	0.663	0.302	1.455	0.305
	Age	0.971	0.936	1.008	0.128
Dysuria	Anti TGA	0.536	0.060	4.825	0.578
	HLA-DQ2	0.394	0.123	1.256	0.115
	HLA-DQ8	66677154.537	0.000		0.998
	Age	1.012	0.946	1.082	0.724
Acyclic pain	Anti TGA	1.727	0.355	8.404	0.498
	HLA-DQ2	1.073	0.644	1.789	0.786
	HLA-DQ8	0.634	0.321	1.252	0.190
	Age	0.994	0.964	1.025	0.694
Infertility	Anti TGA	1.988	0.463	8.534	0.355
	HLA-DQ2	1.067	0.589	1.931	0.831
	HLA-DQ8	0.720	0.328	1.582	0.414
	Age	0.963	0.926	1.002	0.062
Presence of endometriosis	Anti TGA	2.047	0.594	7.058	0.256
	HLA-DQ2	1.303	0.795	2.134	0.294
	HLA-DQ8	0.771	0.372	1.600	0.485
	Age	0.975	0.947	1.004	0.091

a Multiple regression analysis. Anti TGA, Anti-tissue Transglutaminase IgA; OR, Odds Ratio; CI, Confidence Interval.

## Discussion

The aim of this study was to evaluate the relation of HLA-DQ2 and HLA-DQ8 haplotypes that may predispose to CD with endometriosis, its clinical presentation, and the locations of endometriotic implants. Consistent with previous research, there were significant differences between the endometriosis and control groups in terms of symptoms, as pain is a defining characteristic of the disease. A previous study conducted by Fuldeore et al reported similar outcomes in regard to symptoms comparing patients with endometriosis to those without endometriosis.^23^

Although several studies compared endometriosis with HLA-G and HLA-C,^24^, ^25^, ^26^ and some studies compared endometriosis to anti-tissue transglutaminase IgA,^18^,^19^ there have been no reports so far investigating the relation between endometriosis and HLA-DQ2 and HLA-DQ8. On the other hand, studies compared the presence of HLA-DQ2 and HLA-DQ8 markers with other inflammatory conditions, such as Inflammatory Bowel Disease (IBD).^2^,^3^,^27^ In 2018, Bosca-Watts et al. conducted a study quantifying the frequency of CD-related haplotypes in the IBD population, and their findings showed that these haplotypes were not more common in this specific group.^27^ In accordance, these results showed no relation between these markers (analyzed separately or together) with endometriosis, when compared to the control group.

The authors chose the antigens HLA-DQ2 and DQ8 for this study because they represent the antigen presentation for gluten in CD.^5^ The authors hypothesized that CD might have an influence on bowel endometriosis, based on the results of a study that showed the benefits of a gluten-free diet in the progression and symptoms of the disease.^17^ This way, if HLA-DQ2 and DQ8 when expressed, can start an inflammatory chain with gluten as the trigger point,^11^ this hypothesis may be valid. However, the present results showed no statistical relation between HLA-DQ2 and DQ8 and the site or symptoms of endometriosis.

A limitation of this study was the lack of a histopathological diagnosis of CD. Although the present results showed no significant relation between endometriosis and CD, it is still possible that there is a connection between the two that could be confirmed by a histopathological diagnosis. Additionally, the small number of subjects with available anti-tissue transglutaminase IgA results associated with HLA-DQ2 and HLA-DQ8 in the presumptive diagnosis of CD could weaken the statistical analysis. Another possible limitation of this study was its retrospective design. Therefore, a prospective study that evaluates the diagnosis of both CD and endometriosis would provide more reliable results. The strength of this study is its large sample size, which would be difficult to achieve in a prospective cohort.

## Conclusion

This study showed no significant differences in HLA-DQ2 and HLA-DQ8 haplotypes between patients with endometriosis and those without. These haplotypes were not associated with pain symptoms, stage, or site of endometriosis. Further studies with histologically confirmed CD diagnosis are necessary to achieve more accurate conclusions**.**

## Funding

This research did not receive any specific grant from funding agencies in the public, commercial, or not-for-profit sectors.

## Authors’ contributions

Marina P. Andres: Manuscript writing, data collection, data analysis, study design.

Alessandra Peloggia: Manuscript writing, data analysis.

Henrique M. Abrao: Manuscript writing, data collection, data analysis, study design.

Thais F. Magalhaes: Data collection, study design.

João Siufi Neto: Manuscript writing, study design.

Mauricio S. Abrão: Manuscript writing, study design.

## Declaration of Competing Interest

The authors declare no conflicts of interest.
